# High Fiber Cakes from Mediterranean Multipurpose Oilseeds as Protein Sources for Ruminants

**DOI:** 10.3390/ani9110918

**Published:** 2019-11-04

**Authors:** Francesco Serrapica, Felicia Masucci, Emiliano Raffrenato, Maura Sannino, Alessandro Vastolo, Carmela Maria Assunta Barone, Antonio Di Francia

**Affiliations:** 1 Dipartimento di Agraria, Università degli Studi di Napoli Federico II, Via Università 100, 80055 Portici, NA, Italy; francesco.serrapica83@gmail.com (F.S.); maura.sannino@unina.it (M.S.); antonio.difrancia@unina.it (A.D.F.); 2 Department of Animal Sciences, Faculty of AgriSciences, Stellenbosch University, Private Bag X1, 7602 Matieland, South Africa; emiliano@sun.ac.za; 3 Research and Development RUM & N Sas, via Sant’Ambrogio 4/A, 42123 Reggio Emilia, Italy; 4 Dipartimento di Medicina Veterinaria e Produzioni Animali, Università degli Studi di Napoli Federico II, via F. Delpino 1, 80137 Napoli, Italy; alessandro.vastolo@unina.it

**Keywords:** multipurpose oilseed cake, CNCPS fractions, in vitro rumen degradability, rumen bypass protein, sunflower, pomegranate, cardoon, tobacco, hemp

## Abstract

**Simple Summary:**

Recovery and valorization of residues are key factors for agro-industry to progress towards circular-economy models and more sustainable productions. In the vegetable oils industry, large quantities of spent seed cakes are produced downstream of the oil extraction processes, and their use as animal feedstuffs, mainly as protein supplements for ruminants, is a possible valorization strategy. In this study, we analyzed chemical composition and in vitro digestibility of spent cakes from sunflower, pomegranate, cardoon, tobacco and hemp that are multipurpose cultures emerging in Mediterranean area. The results showed that the cakes of tobacco, cardoon and hemp might be interesting alternative protein feeds for ruminants. The valorization of these cakes may potentially improve economic and environmental sustainability of the emerging vegetable-oil production chains.

**Abstract:**

Fifteen oilseed cakes from sunflower, pomegranate, cardoon, tobacco and hemp were characterized with regard to chemical composition, Cornell Net Carbohydrate and Protein System (CNCPS) fractionation, in vitro digestibility of dry matter, neutral detergent fiber, and crude protein. All the cakes presented low moisture, rather variable ether extract contents and medium to high levels of crude protein and neutral detergent fiber. The cakes significantly differed in terms of CNCPS partitioning and in vitro digestibility. Tobacco and hemp cakes presented high contents of slow degradable fractions of crude protein and carbohydrate joined to good post-ruminal protein digestibility. Cardoon cakes presented the highest rumen protein degradability. Based on crude protein content and intestinal digestibility of rumen undegraded protein, cakes of tobacco and hemp showed the better potential as alternative protein supplements for ruminants, while pomegranate appears to be the least suitable for ruminant feeding.

## 1. Introduction

As a result of production intensification, the current agri-food sector in developed countries is characterized by considerable materials’ consumption and waste streams to be disposed, so that it can be configured according to a model of linear economy [1]. Hence, the implementation of strategies to support the transition toward a circular economy model balancing economic development with environmental and resource protection [2] is a relevant issue for the whole agri-food sector [3,4]. In the Mediterranean area, the protein needs of ruminants are mainly supplied by soybean meal imported from elsewhere [5,6,7]. On the other hand, emerging agro-industrial multi-purpose crops such cardoon (*Cynara cardunculus* L.) and hemp (*Cannabis sativa* L.) along with well-established crops, mainly smoking tobacco (*Nicotiana tabacum* L.) and pomegranate (*Punica granatum* L.) have found additional use for oil production in recent years. A common feature of these crops is the relatively low requirement in terms of cultivation inputs [8,9,10]. Cardoon and hemp oils can be used for different purposes such human consumption, biodiesel production and as traditional medicine ingredient [11,12,13]. Similarly, tobacco oil finds use as a raw material in manufacturing soaps, paints, alkyd resins, lubricants, fuel, besides as edible nicotine-free oil [14,15]. Oil extracted from pomegranate seeds left over from the juices preparation is mainly used in food, pharmaceutical, and cosmetic industries [16,17]. 

Oilseed cakes resulting from mechanical extraction of oil may contain remarkable levels of protein, but also varying amounts of fiber [18,19]. The cakes from these emerging oilseeds might then be used as protein feeds for ruminants, so allowing both to exploit residues from oil extraction and to reduce the ecological footprint of livestock production [20,21]. As far we know, there are no data on nutritional characteristic of cakes from tobacco and pomegranate seeds, whereas the studies regarding cardoon cakes [22,23] have not evaluated availability of crude protein throughout the whole digestive tract. Although data are available for hemp cakes, they refer to northern-Europe varieties, which differ in terms of botanical and agronomic traits compared to those cultivated in Mediterranean area [24,25]. Therefore, this study aimed at characterizing oilseed cakes from cardoon, hemp, tobacco and pomegranate with respect to chemical composition and in vitro digestibility.

## 2. Materials and Methods 

### 2.1. Oilseed Cake Sampling and Chemical Analyses

Residual cakes from after oil extraction from seeds of pomegranate (PoC), tobacco (ToC), cardoon (CaC), and hemp (HeC) were obtained at a commercial plant located in Campania Region, southern Italy. For each cake type, three samples (0.5 kg each) were gained after oil extraction from different batches of seeds produced during the 2017 harvest campaign. The conditions of extraction were the following: nozzle diameter of screw press, 8 mm; screw rotation speed, 22 rpm; seeds pre-treatment T, 50 °C; oil extraction T, 70 °C. Hereafter, these cakes will be referred as emerging cakes. In addition, three samples of sunflower cake (SuC) were collected at a commercial feed manufacturer intended to be used for comparison, and they will be denoted as reference cake. The samples were dried (65° C in a ventilated oven), ground in a laboratory mill (Brabender Wiley mill, Brabender OHG Duisburg, Germany) by using a 1-mm mesh (2-mm for in vitro protein disappearance) and stored at 4 °C until the analyses, which were performed within 3 weeks. The cakes derived from extraction of oil for human consumption, except ToC from biofuel production, and PoC from cosmetic oil production. 

The 15 samples were assayed according to Association of Official Analytical Chemists [26] for dry matter (DM; method 930.15), ash (method 942.05), crude protein (CP; method 976.05) and ether extract (EE; method 954.02). The organic matter (OM) content was calculated as the difference between DM and ash contents, with ash determined by combustion at 550 °C overnight. Neutral detergent fiber (NDF) and acid detergent fiber (ADF) were determined according to the method of Van Soest et al. [27] using an Ankom^220^ Fiber Analyzer unit (Ankom Technology Corporation, Fairport, NY, USA). Sodium sulphite and heat-stable amylase (activity 17.400 Liquefon units/mL, Ankom Technology) were used in the NDF procedure. Acid detergent lignin (ADL) was determined by treating the ADF residue with 72% sulphuric acid [28]. All fiber fractions were expressed exclusive of residual ash. The starch content was determined by polarimetry (Polax 2L, Atago, Tokyo, Japan) according to Ewers’ method as described by the standard ISO 6493 [29]. The soluble protein (SP), non-protein nitrogen (NPN), protein bound to NDF (insoluble neutral-detergent protein, NDIP) and to ADF (acid-detergent insoluble protein, ADIP) were determined according to the procedure recommended by Licitra et al. [30]. Each analysis was performed at least in duplicate. The content of metabolizable energy (ME) were estimated according to the INRA (Institute National de la Recherche Agronomique) energy system [31].

### 2.2. Protein and Carbohydrate Fractionation

The Cornell Net Carbohydrate and Protein System (CNCPS) version 6.5 was used to fractionate and characterize proteins (PA, PB1, PB2, PB3, PC) and carbohydrates (CA, CB1, CB2, CC) according to Van Amburgh et al. [32]. As regards protein, the PA fraction (calculated as NPN × 6.25) is assumed to be very rapidly degraded in the rumen. The fractions PB1 (SP minus PA), PB2 (100 minus SP and NDIP), and PB3 (NDIP minus ADIP) represent the fast, medium and slow degradable protein, respectively. The fraction PC designates the unavailable protein and corresponds to ADIP. As regards carbohydrates (CHO), the total carbohydrates (TC) were calculated as 100 minus CP, EE and ash; the structural carbohydrates (SC) as NDF minus NDIP; the non-structural carbohydrates (NSC) as TC minus SC. The CNCPS carbohydrates fractions were calculated as follows:

CA (g/kg TC) = [1000 − starch (g/kg NSC)] × [1000 − B2 (g/kg TC) − C (g/kg TC)]/1000

CB1 (g/kg TC) = starch (g/kg NSC) × [1000 − B2 (g/kg TC) − C (g/kg TC)]/1000

CB2 (g/kg TC) = 1000 × [NDF (g/kg DM) − NDPI (g/kg CP) × 0.001 × CP (g/kg DM) − NDF (g/kg DM) × 0.001 × Lignin (g/kg NDF) × 2.4]/TC (g/kg DM)

CC (g/kg TC) = 1000 × [NDF (g/kg DM) × 0.001 × Lignin (g/kg NDF) × 2.4]/TC (g/kg DM).

The fraction CA, mainly composed of sugars and short oligosaccharides, represents the readily, water-soluble carbohydrates; the fraction CB1, contains starch and non-starch (pectines, beta-glucans, galactans and gums) polysaccharides soluble in neutral detergent solution, and represents the medium degradable carbohydrates; the fraction CB2 contains the digestible cell wall and represents the slow degradable carbohydrates; the fraction CC contains cell walls and lignin and represents the undegradable and indigestible SC.

### 2.3. In Vitro Study

#### 2.3.1. Dry Matter and NDF Digestibility

In vitro digestibility of DM (IVDMD) and NDF (IVNDFD) were determined in a Daisy II system (Ankom, Tech. Co., Fairport, NY, USA) by the procedure by Robinson [33] based on a 48-h incubation at 39 °C in presence of buffered rumen fluid, as described by Uzun et al. [34]. Three filter bags/sample (Ankom F57, Ankom Technology Corp., Fairport, NY, USA) were filled with 250 mg of milled sample (1 mm). Three digestion jars (4 L capacity) were filled with pre-warmed buffer solution consisting of mixture of solution A (1330 mL/incubation jar) and B (266 mL/incubation jar). The solution A was prepared dissolving in H_2_O 10.00 g/L of KH_2_PO_4_, 0.50 g/L of MgSO_4_ 7H_2_O, 0.50 g/L of NaCl, 0.10 g/L of CaCl_2_ 2H_2_O and 0.50 g/L of urea (reagent grade), whereas solution B consisted of 15.00 g/L of Na_2_CO_3_ and 1.00 g/L of Na_2_S-9H_2_O. Before the start of incubation, each jar was vertically divided by using perforated plastic divider, filled with the combined buffer solutions, and left in the incubator at 39 °C for at least 2 hours. Ruminal fluid was collected post-mortem from four young bulls at a local abattoir (C.S.M. SOC. COOP. A.R.L., Pompei, Italy), by 3 min postmortem. At slaughtering time, animals were fasted overnight, but allowed free access to water and straw throughout, then weighed, stunned by a penetrating captive bolt device and slaughtered in accordance to the EU Regulation 2009/1099/EC. Donor bulls were selected at a fattening cattle farm before slaughtering and they were fed a diet containing soybean meal as the main protein source. In the abattoir, the reticulo-rumen of each animal was incised to form a fairly wide opening enough to gain content access and, at same time, to prevent it from leaking out. The rumen content was collected from at least four different locations, roughly filtered to remove larger particles, and immediately placed into pre-warmed (39 °C), 2-L airtight glass-bottles filled with carbon dioxide (CO_2_). The rumen fluid was used by 20–25 min from the collection. In the lab, rumen fluids were mixed, filtered through two layers of cheesecloth under constant flushing of CO_2_ and transferred in the jars (400 mL/jar) along with the filled filter bags. Three bags/sample were placed into different jars, for a total of 16 bags for jar with 8 bags on each internal divider’ side. After 48 h of incubation at 39 °C, the bags were removed, washed in cold tap water until the wash water was clear, and analyzed for NDF content.

#### 2.3.2. Crude Protein Disappearance

The rumen undegradable protein (RUP) and intestinal digestibility of RUP (IDRUP) were estimated by the in vitro two-steps procedure described by Ross et al. [35]. Briefly, 40 mL of pre warmed Van Soest’ ruminal buffer [36] and 10 mL of rumen fluid, obtained as previous described, were added to an Erlenmeyer flask (125 mL) containing 500 mg of sample (2 mm grid). Four flasks were prepared for each sample. The flasks sealed with rubber stoppers were simultaneously incubated at 39 °C in shaking water baths (shaking frequency of 100 rpm) under continuous carbon dioxide flow. For the ruminal step, after 16 h of incubation, the contents of two flasks/sample were vacuum filtered with boiling water through a previously calibrated filter (Whatman 934AH). For the intestinal step, in the remaining flasks, 3 M HCl was added until a pH value of about 1.9, followed after 1 min by 2 mL of pepsin solution (282 U/mL; Pepsin P-7000, Sigma-Aldrich, St. Louis, MO, USA). After 1 h of incubation, 2 mL of NaOH was added to neutralize the medium and stop the pepsin reaction, followed by 10 mL of a buffered enzymatic mixture (1.8 M KH_2_PO_4_ buffer) containing trypsin (24 mg/mL; Trypsin T4799, Sigma-Aldrich), chymotrypsin (20 mg/mL; α-Chymotrypsin type II, C4129, Sigma-Aldrich), amylase (50 mg/L; α-Amylase type I-A, A6255, Sigma-Aldrich) and lipase (4 mg/mL; Lipase type VI-S, L0382, Sigma-Aldrich). After a further 24 h incubation, the flask content was filtrated. The residues from the two steps were water-washed (four time with 25 mL of boiling distiller water), allowed to air dry and were analyzed for CP. The RUP and IDRUP contents were estimated as the ratio between the recovered undegraded CP after the ruminal and intestinal steps and the corresponding CP of the sample material. Rumen degraded crude protein (RDP) was calculated as CP—RUP, while intestinal digestible CP (IDCP) was determined as RUP × IDRUP. Total digestibility of CP (TDCP) was calculated as the sum of RDP and IDCP.

### 2.4. Statistical Analyses

Statistical analysis was performed by using SAS package software version 8.1 (SAS Institute, Cary, NC, USA). The CNCPS fractions and the in vitro determinations were analyzed by one-way ANOVA, GLM procedure, by using the cake type as the statistical unit and according to the following model:

y_ij_ = μ + Ca_i_ + ε_ij_

where y is the experimental data, μ is the general mean, Ca is the substrate (i = 1, 2, …, 5), and ε is the error term. The values below detectable levels were considered as missing values. A Bonferroni Multiple Comparison of Means test was used to determine significance of the main effect. In addition, Pearson correlation was performed between the data from in vitro study and the CNCPS fractions. Significance was declared at *p* < 0.05.

## 3. Results

The chemical composition of both the reference and the emerging cakes is shown in Table 1. The moisture and the ash levels were similar within and among the cakes and averaged below 100 g/kg DM. By contrast, the EE amounts were rather variable, by ranging from less than 10 g/kg DM (PoC) to a maximum of 158 g/kg DM observed for a sample of ToC. Almost all the cakes presented CP contents over 200 g/kg DM, and NDF and ADF levels varying around 400–500 g/kg DM and 300–400 g/kg DM, respectively. By contrast, the PoC cakes had lower CP and higher NDF and ADF levels. The ADL contents were variable among and within the samples and averaged above 100 g/kg DM except HeC (63.1 g/kg DM) and CaC (64.3 g/kg DM). The starch levels were always less than 100 g/kg DM and for ToC were below the detection limit. The ME of the emerging cakes ranged from 5.94 (PoC) to 9.05 MJ/kg DM (ToC), this latter value being comparable to those of SuC (9.56 MJ/kg DM).

The Table 2 shows the protein and carbohydrate fractions evaluated according the CNCPS system. Compared to the reference cake SuC, all emerging cakes presented significantly lower values of the protein fraction PA, which were not detected in PoC. The PB2 was the largest fraction for all the cake, except CaC that presented more than half the CP included in the PB1 fraction. The unavailable protein PC was rather similar between the cakes, except the higher values (*p* < 0.05) of PoC and CaC compared to SuC. Overall, against an unavailable protein content rather similar between cakes, the CaC and PoC cakes, and in minor extent ToC, presented a higher proportion of medium-slow fermentation fractions (PB2 + PB3), whereas opposite trend was observed for CaC. The sole SuC showed comparable levels of fast (PA + PB1) and medium-slow degradable protein. In regard to carbohydrates, the emerging cakes presented higher (*p* < 0.05) structural carbohydrates and, consequently, lower NSC levels compared to SuC. The sole CaC samples showed SC and NSC values comparable to SuC. In terms of degradability, the emerging cakes had lower (*p* < 0.05) values of the CA fraction, except CaC, and of the unavailable fraction CC, except ToC and PoC, while, as expected, those of the CB2 fraction were higher (*p* < 0.05). No differences were found among the cakes for the CB1 fraction. Overall, the unavailable cell wall fraction (CC) largely differed between cake being, for example, more than double in ToC compared to HeC and CaC as an effect of the very high contents of both NDF and ADL contents. The slowly degradable fractions (CB1 + CB2) exceed the ready fraction CA in all the cakes except SuC, which showed comparable values of fast and slow degradable carbohydrate fractions. 

The Table 3 shows the results of the in vitro studies. The highest (*p* < 0.05) value of RUP were observed for PoC and HeC, followed in the order by ToC, CaC, and SuC. Consistently, HeC showed the highest (*p* < 0.05) value of IDP, followed by ToC and PoC and finally CaC and SuC. The values from ruminal and intestinal steps resulted in the highest (*p* < 0.05) TTPD values observed for ToC and SuC followed in the order by HeC, CaC and PoC. The rumen degradability of DM and especially of NDF of the emerging cakes were quite low especially for PoC. The highest (*p* < 0.05) values of IVDMD were detected for the ToC samples, followed in the order by SuC, CaC, HeC and PoC. A similar trend was observed for IVNDFD, but no differences were observed among CaC, HeC and PoC.

The correlation coefficients between the CNCPS fractions and in vitro digestibility are in Table 4. The RDP correlated positively to the fraction PA of protein (*p* < 0.001), and fraction CC of carbohydrates (*p* < 0.05), and negatively to the fraction CB2 of carbohydrates (*p* < 0.001) and fraction PC of protein (*p* < 0.01). The IDP correlated negatively to the fraction PA of protein (*p* < 0.01). The IVDDM correlated negatively to the fraction CB2 of carbohydrates and PC of protein (*p* < 0.01), and positively to CC (*p* < 0.01) of carbohydrates. Similarly, IVDNDF correlated negatively to fraction CB2 (*p* < 0.01) and positively to fraction CC (*p* < 0.001) of carbohydrate.

## 4. Discussion

Large quantities of crop residues and agro-industrial by products are produced worldwide every year and, besides the presence of undesirable substances, three of these are the main features potentially limiting their use as livestock feeds [37]. Firstly, seasonal production that, when coupled with high moisture content, reduce their temporal and spatial availability [38]. Secondly, low concentration of nutritive components that can negatively affect animal performances [39]. Thirdly, large compositional variability, related to different vegetable raw material and industrial processes that can make difficult diet formulation. Thus, important features of the emerging cakes are the moisture and ash levels less than 100 g/kg DM that allow for easy storage and transport and do not penalize the energy content. In addition, the chemical composition was rather constant, except EE and ADL, since the cakes derived from oilseed crops still little cultivated so that few cultivars are available and were obtained from the same factory under similar oil extraction conditions.

Except PoC, the cakes contained noticeable amounts of lipids originated from the oil residual after the mechanical extraction that contributes to their good ME content (on average 8.1 MJ/kg DM). The EE levels of HeC and CaC were in agreement with the values reported by others [22,23,40,41], while no reports are available about PoC and ToC composition. The reduced fat content of PoC is related to the small amount of oil in pomegranate seeds [42,43]. On the other hand, high level of fat, as is for ToC, HeC and CaC, can affect storability and safety of the feeds due to secondary products from lipid oxidation [44] and to development of molds [45]. Nevertheless, literature indicates that fatty acid composition of oils from hemp, cardoon and tobacco, as most oilseeds, are characterized by a high unsaturation degree, with predominance of α-linolenic acid in hemp and linoleic acid in cardoon and tobacco [46,47,48]. Thus, since the fatty acids profile of ruminant derived foods largely depends on dietary factors, the use of these cakes might increase the content of health-promoting fatty acids in milk and meat [49,50,51,52]. 

Except PoC, the CP contents of the emerging cakes were higher or comparable to that of the reference sunflower cakes, so that they can be classified as medium-high protein feeds. As seen for the lipid contents, the CP concentrations of HeC and CaC were similar to those reported by others [22,23,40,41]. In terms of CNCPS partitioning of CP and CHO, SuC presented comparable values of the fractions at fast and slow degradation rate, ToC, HeC, PoC were imbalanced towards the slow degradable fractions, and CaC presented higher fractions of fast degradable CP and slow degradable CHO. It follows that diets formulated only on the basis of CP content of these cakes can unbalance the protein supply to the ruminants [53].

In regard to the CP in vitro digestibility, consistently with the data of CNCPS fractionating, CaC showed the highest RDP value except SuC. A comparable RDP value was reported for cardoon cakes by Cabiddu et al. [23] through same in vitro procedure. The low RDP value observed for HeC agrees with the CNCPS data and the results previously observed in vitro on hemp cake, and in situ on hemp meal [24,54]. The rather low ruminal CP disappearance of HeC was compensated by post-ruminal digestion resulting in a TTDP value comparable, although slightly lower, to those of SuC and ToC. By contrast, PoC had low values of both RDP and IDP. These differences may be related to the different contents in soluble N fractions and fiber-associated proteins highlighted in the CNCPS partitioning. To confirm this, RDP correlated (*p* < 0.001) positively to PA and negatively to PC, even if others did not observe a direct effect of ADIP level on protein availability [55,56,57]. Reduced rumen degradability is a typical trait of heat-treated vegetable proteins, mainly due to heat-promoted peptide chain cross-linkage rearrangements and chemical bounds with carbohydrates [58]. In lack of heat treatment, the higher estimated RUP content observed for ToC, HeC and PoC in comparison to SuC and CaC can be likely due to differences in the source material, including the presence of gelatinous compounds and lack of surface area for bacterial to adhere [59,60].

The very low values of NDF degradability was significantly and negatively correlated with the slow degradable fraction of carbohydrate CB2. An apparently contradictory positive correlation between IVDNDF and the unavailable fiber CC was found. The CC fraction indicates the cellulose lignification degree, and indeed as cell wall lignification increases, cellulose fiber become more fragile with formation of smaller, more compact, easily hydratable particles more prone to microbial attack and degradability [61,62].

Finally, the results allowed us to outline the potential use of the emerging cakes in ruminant feeding. In regard to PoC, the CP content rather limited and characterized by a low TDDP value joined to the very high ADL and unavailable fiber contents make this by-product not very suitable for ruminant feeding. The cakes CaC and SuC, obtained from oilseeds belonging to the same botanical family, showed similar characteristics in term of chemical composition, protein content and degradability, so that their dietary use may be similar. The relatively high digestible RUP of ToC and HeC can play a role in diets for high producing dairy cows, especially when fresh roughages and legumes are utilized so that the basal diet may be deficient in RUP [63]. Moreover, on average, ToC and HeC presented higher CP contents than SuC, resulting in overall potentially better protein sources.

## 5. Conclusions

On the basis of the analytical data and in vitro determinations, the cakes residual after oil extraction from cardoon, hemp, and tobacco seeds may be potentially used as protein feeds for ruminants. Alternative uses have to be identified for residues from pomegranate seeds due to the limited protein content joined to the very high unavailable carbohydrate levels. 

## Figures and Tables

**Table 1:** Chemical composition (g/kg dry matter, DM, if not otherwise stated) of the oilseed cakes (mean and standard deviation of 3 different cake samples).

Item	Overall Mean	SD	Oilseed Cake				
			SuC (n = 3)	PoC (n = 3)	CaC (n = 3)	ToC (n = 3)	HeC (n = 3)
Dry Matter (g/kg)	914.5	6.92	907.1 ± 7.4	915.5 ± 0.1	923.9 ± 1.5	907.3 ± 1.7	918.7 ± 5.0
Ash	57.9	10.9	65.7 ± 3.2	41.0 ± 2.2	55.7 ± 2.9	56.5 ± 5.7	70.7 ± 9.0
Crude Protein	243.8	81.2	199.3 ± 30.0	149.1 ± 3.5	211.3 ± 10.6	369.7 ± 17.3	289.7 ± 37.6
Ether Extract	89.7	48.5	139.6 ± 14.6	9.5 ± 2.4	77.2 ± 5.3	120.4 ± 33.5	102.0 ± 34.9
NDF	514.5	111.0	411.0 ± 63.0	715.7 ± 7.8	467.9 ± 22.0	467.2 ± 39.6	518.8 ± 43.3
ADF	379.5	68.3	326.3 ± 28.6	505.3 ± 6.6	359.7 ± 16.3	347.0 ± 32.7	395.5 ± 21.3
ADL	90.5	25.3	107.0 ± 20.8	108.8 ± 3.7	64.3 ± 25.0	109.5 ± 9.0	63.1 ± 23.5
Starch	44.9	26.3	63.7 ± 18.5	57.9 ± 4.0	46.7 ± 13.0	ND	56.2 ± 41.0
NPN (g/kg CP)	45.1	62.9	165.0 ± 70.1	ND	17.8 ± 6.8	24.0 ± 6.2	18.7 ± 4.5
Soluble Protein	287.7	188.3	411.7 ± 136.5	31.6 ± 13.6	539.0 ± 23.3	310.3 ± 10.5	145.7 ± 94.2
NDIP	163.2	26.6	140.0 ± 26.9	205.0 ± 20.0	158.3 ± 1.5	150.3 ± 6.4	162.3 ± 9.6
ADIP	134.9	20.8	103.7 ± 29.4	147.7 ± 20.4	153.7 ± 2.3	130.7 ± 3.2	139.0 ± 9.1
ME (MJ/kg DM)	8.1	1.4	9.56 ± 0.1	5.94 ± 0.4	8.26 ± 0.4	9.05 ± 0.5	7.49 ± 0.7

NDF, neutral detergent fiber; ADF, acid detergent fiber; ADL, acid detergent lignin; NPN, non-protein nitrogen; NDIP, insoluble neutral detergent protein; ADIP, acid detergent insoluble protein; ME, metabolizable energy; SuC, sunflower cake; PoC, pomegranate cake; ToC, tobacco cake; CaC, cardoon cake; HeC, hemp cake; n = number of samples; ND, not detected.

**Table 2:** Crude protein and carbohydrate fractions of the oilseed cakes according the Cornell Net Carbohydrate and Protein System (CNCPS) system (LSM).

Item	Oilseed Cake					SEM	*p* Value
	SuC (n = 3)	PoC (n = 3)	CaC (n = 3)	ToC (n = 3)	HeC (n = 3)		
Protein fractions (g/kg CP)							
PA	165.0 ^a^	ND	17.8 ^b^	24.0 ^b^	18.7 ^b^	7.07	<0.0001
PB1	246.7 ^b^	31.6 ^d^	521.2 ^a^	286.3 ^b^	127.0 ^c^	13.67	<0.0001
PB2	448.3 ^d^	763.4 ^a^	302.7 ^e^	539.3 ^c^	692.0 ^b^	21.42	<0.0001
PB3	36.3 ^ab^	57.3 ^a^	4.7 ^c^	19.7 ^b^	23.3 ^ab^	11.65	0.0053
PC	103.7 ^b^	147.7 ^a^	153.7 ^a^	130.7 ^ab^	139.0 ^ab^	14.28	0.019
Carbohydrates (g/kg DM)							
TC	595.4 ^bc^	800.4 ^a^	655.8 ^b^	453.4 ^d^	537.6 ^c^	20.25	<0.0001
SC	383.1 ^b^	685.1 ^a^	434.4 ^b^	411.7 ^a^	464.1 ^a^	39.10	<0.0001
NSC	212.3 ^a^	115.3 ^b^	221.5 ^a^	41.7 ^c^	73.5 ^bc^	23.27	<0.0001
Carbohydrate fractions (g/kg TC)							
CA	249.5 ^a^	71.7 ^b^	265.6 ^a^	91.5 ^b^	31.2 ^b^	45.50	0.0006
CB1	107.0	72.3	71.3	ND	105.7	26.54	0.26
CB2	212.4 ^d^	529.8 ^ab^	426.1 ^bc^	328.7 ^c^	581.9 ^a^	36.76	<0.0001
CC	431.1 ^ab^	326.2 ^bc^	237.0 ^c^	579.8 ^a^	281.2 ^bc^	52.58	0.0003

PA, non-protein nitrogen; PB1, true soluble protein; PB2, neutral detergent soluble protein; PB3, neutral detergent insoluble protein but soluble in acid detergent; PC, insoluble protein in acid detergent; TC, total carbohydrate; SC, structural carbohydrate; NSC, non-structural carbohydrate; CA, sugars and soluble fraction; CB1, starch and non-starch polysaccharides soluble in neutral detergent; CB2, fraction available cell wall; CC, unavailable cell wall. SuC, sunflower cake; PoC, pomegranate cake; ToC, tobacco cake; CaC, cardoon cake; HeC, hemp cake. n = number of samples. ND, not detected. The means in a row with different superscripts differ (*p* < 0.05); LSM, least square mean; SEM, standard error of means.

**Table 3:** In vitro digestibility of crude protein, dry matter, neutral detergent fiber (LSM) of the oilseed cakes.

Item	Oilseed Cake					SEM	*p* Value
	SuC (n = 3)	PoC (n = 3)	CaC (n = 3)	ToC (n = 3)	HeC (n = 3)		
Crude protein disapparence							
RDP (g/kg CP)	691.8 ^a^	283.2 ^e^	625.8 ^b^	495.3 ^c^	329.3 ^d^	5.51	<0.0001
RUP (g/kg CP)	308.2 ^d^	716.8 ^a^	374.2 ^d^	504.7 ^c^	670.7 ^b^	5.52	<0.0001
IDRUP (% RUP)	12.1 ^b^	13.2 ^b^	14.3 ^b^	48.9 ^a^	56.0 ^a^	2.66	<0.0001
IDP (g/kg CP)	37.3 ^d^	94.6 ^c^	53.1 ^d^	246.4 ^b^	375.6 ^a^	12.89	<0.0001
TTDP (g/kg CP)	729.1 ^ab^	377.8 ^d^	678.9 ^c^	741.5 ^a^	705.3 ^b^	11.79	<0.0001
Rumen digestibility							
IVDMD (% DM)	76.0 ^b^	44.5 ^e^	65.0 ^c^	81.0 ^a^	61.2 ^d^	1.26	<0.0001
IVNDFD (% NDF)	35.0 ^b^	14.0 ^c^	17.4 ^c^	54.6 ^a^	15.5 ^c^	3.02	<0.0001

CP, Crude protein; RDP, rumen degradable protein; RUP, rumen undegradable protein; IDRUP, intestinal digestibility of RUP; IDP, intestinal digested protein; TTDP, total tract digested protein; IVDMD, in vitro dry matter (DM) degradability; IVNDFD, in vitro neutral detergent fiber (NDF) degradability. SuC, sunflower cake; PoC, pomegranate cake; ToC, tobacco cake; CaC, cardoon cake; HeC, hemp cake. *n* = number of samples. The means in a row with different superscripts differ (*p* < 0.05); LSM, least square mean; SEM, standard error of mean.

**Table 4:** Correlation coefficients between in vitro digestibility and CNCPS Carbohydrate and Protein fractions in the oil-seeds cakes.

Item	Carbohydrate Fractions				Protein Fractions				
	CA	CB1	CB2	CC	PA	PB1	PB2	PB3	PC
RDP	0.40	0.20	−0.83 ***	0.54 *	0.91 ***	−0.09	−0.17	0.38	−0.73 **
IDP	−0.40	0.09	0.51	−0.18	−0.89 ***	0.18	0.04	−0.48	0.39
IVDMD	0.32	0.42	−0.75 **	0.64 **	0.16	−0.14	0.1	−0.11	−0.72 **
IVNDFD	0.01	0.40	−0.69 **	0.93 ***	0.34	−0.1	−0.26	0.37	0.04

CP, Crude protein; RDP, rumen degradable protein; RUP, rumen undegradable protein; IDRUP, intestinal digestibility of RUP; IDP, intestinal digested protein; TTDP, total tract digested protein; IVDMD, in vitro dry matter (DM) degradability; IVNDFD, in vitro neutral detergent fiber (NDF) degradability. SuC, sunflower cake; PoC, pomegranate cake; ToC, tobacco cake; CaC, cardoon cake; HeC, hemp cake. * *p* < 0.05; ** *p* < 0.01; *** *p* < 0.001.
